# Liposomal Delivery of the RNA Genome of a Live-Attenuated Chikungunya Virus Vaccine Candidate Provides Local, but Not Systemic Protection After One Dose

**DOI:** 10.3389/fimmu.2020.00304

**Published:** 2020-03-05

**Authors:** Eranga Abeyratne, Kothila Tharmarajah, Joseph R. Freitas, Helen Mostafavi, Suresh Mahalingam, Ali Zaid, Mehfuz Zaman, Adam Taylor

**Affiliations:** ^1^The Emerging Viruses, Inflammation and Therapeutics Group, Menzies Health Institute Queensland, Griffith University, Southport, QLD, Australia; ^2^Institute for Glycomics, Griffith University, Southport, QLD, Australia

**Keywords:** chikungunya, vaccine, liposome, RNA, live-attenuated

## Abstract

Chikungunya virus (CHIKV) is the causative pathogen of chikungunya fever, a mosquito-borne viral disease causing highly debilitating arthralgia that can persist for months and progress to chronic arthritis. Our previous studies have identified the CHIKV live-attenuated vaccine candidate CHIKV-NoLS. Like most live-attenuated vaccines, attenuated replication of CHIKV-NoLS has the potential to limit scalable production. To overcome production limits, as well as other drawbacks of live-attenuated vaccines, we developed an *in vivo* liposome RNA delivery system to deliver the self-replicating RNA genome of CHIKV-NoLS directly into mice, allowing the recipients' body to produce the live-attenuated vaccine particles. CAF01 liposomes were able to deliver replication-competent CHIKV-NoLS RNA *in vitro*. Immunodeficient AG129 mice inoculated with liposome-delivered CHIKV-NoLS RNA developed viremia and disease signs representative of this lethal model of CHIKV infection, demonstrating *de novo* vaccine particle production *in vivo*. In immunocompetent C57BL/6 mice, liposome-delivered CHIKV-NoLS RNA inoculation was associated with reduced IgM and IgG levels with low antibody CHIKV-neutralizing capacity, compared to vaccination with the original live-attenuated vaccine CHIKV-NoLS. One dose of liposome-delivered CHIKV-NoLS RNA did not provide systemic protection from CHIKV wild-type (WT) challenge but was found to promote an early onset of severe CHIKV-induced footpad swelling. Liposome-delivered CHIKV-NoLS RNA inoculation did, however, provide local protection from CHIKV-WT challenge in the ipsilateral foot after one dose. Results suggest that in the presence of low CHIKV-specific neutralizing antibody levels, local inflammatory responses, likely brought on by liposome adjuvants, have a role in the protection of CHIKV-induced footpad swelling in the ipsilateral foot of mice inoculated with liposome-delivered CHIKV-NoLS RNA. Low IgG and CHIKV-specific neutralizing antibody levels may be responsible for early onset of severe swelling in the feet of CHIKV-WT-challenged mice. These results support previous studies that suggest CHIKV is vulnerable to antibody-mediated enhancement of disease. Further studies using booster regimes aim to demonstrate the potential for liposomes to deliver the self-replicating RNA genome of live-attenuated vaccines and offer a novel immunization strategy.

## Introduction

Chikungunya fever is a mosquito-borne viral disease caused by the chikungunya virus (CHIKV). CHIKV is a positive-sense single-stranded RNA (+ssRNA) virus and has emerged as a major global human pathogen in the twenty-first century as its geographic distribution continues to expand. Epidemics have been reported in the Indian Ocean islands of Mauritius, La Réunion, and the Seychelles (270,000 cases in La Réunion in 2005–2006) and India (3–4 million estimated cases in 2005–2011) (1–3). CHIKV has recently become established in the Americas, infecting over 1.5 million people in just 2 years (4). The majority of individuals infected with CHIKV develop disease (5, 6). Symptoms of chikungunya disease are characterized by the acute onset of fever and joint pain lasting 7–10 days. Crippling arthritic symptoms usually involve multiple joints in a symmetric distribution. A high proportion of patients experience prolonged articular pain and arthritis lasting several weeks to years (7). This chronic form of CHIKV disease contributes considerably to disease burden. Atypical presentations associated with CHIKV infection include hepatitis, Guillain-Barré syndrome, myocarditis, retinitis, and nephritis (8). Increases in mortality have been described in several CHIKV epidemics with most deaths occurring in neonates, patients with underlying medical conditions, and older persons (9–11). To date, no safe commercial vaccine is available.

Our previous studies report the development of a live-attenuated CHIKV vaccine candidate CHIKV-NoLS (12, 13). Mutating the nucleolar localization sequence (NoLS) of CHIKV capsid protein, replacing 10 wild-type (WT) amino acids with alanines, significantly attenuates virus replication (12). CHIKV-NoLS is highly attenuated *in vivo*, and mice immunized with one dose of CHIKV-NoLS are fully protected from subsequent challenge with CHIKV-WT (12). Additionally, this vaccine shows cross-protection against other arthritogenic alphaviruses (12). A live-attenuated vaccine is a desirable means of disease prevention due to ease of production, low cost, and avoidance of multiple boosters. However, large-scale propagation of CHIKV-NoLS may be limited by its attenuated replication, a common hurdle for the development of many live-attenuated vaccines. The modifications that make CHIKV-NoLS safe and effective for use as a vaccine prevent rapid, large-scale production of the vaccine using traditional vaccine substrates. An alternative vaccine delivery vehicle that removes the need for *in vitro* scale-up would remove the production limit on CHIKV-NoLS. RNA has emerged as an effective platform to safely and cheaply deliver vaccines using nanoparticle delivery vehicles such as liposomes (14).

Liposomes are vesicles consisting of phospholipid bilayers. They can be readily manufactured with various lipid compositions in different particle sizes and charges, allowing the tailored design of formulations that are optimal for the delivery of a biomolecule of interest (15, 16). Liposomes have shown potential for the delivery of various therapeutics *in vivo* including siRNA, miRNA, DNA, and peptides (14, 17). CAF01 is a liposome composed of a cationic quaternary ammonium salt adjuvanted with a glycolipid immunomodulator (18). Recent work with tuberculosis and malaria antigens has shown that CAF01-delivered vaccines are able to induce good cellular and antibody responses (19, 20). Safety, tolerability, and immunogenicity of CAF01 has been tested in five phase I clinical trials in combination with four different peptide antigens including a tuberculosis vaccine candidate and an HIV-1 peptide cocktail (21).

Upon entry into cells, the +ssRNA genome of alphaviruses, like mRNA, is rapidly translated by the host machinery to produce the viral proteins required for RNA synthesis and virus replication. In this study, we pioneer the use of CAF01 to deliver the +ssRNA genome of CHIKV-NoLS *in vivo*. Using this novel immunization strategy, we observe *de novo* production of live-attenuated vaccine *in vivo*. Immunization of mice with one dose of CAF01-delivered CHIKV-NoLS RNA produced CHIKV neutralizing antibodies and provided regional protection from disease following CHIKV challenge. This study provides the basis for developing CAF01-mediated delivery of replication-competent RNA as a vaccine strategy. Further characterization of the live-attenuated CHIKV vaccine candidate CHIKV-NoLS demonstrates the high degree of attenuation *in vivo* and potent immunogenicity after one dose.

## Materials and Methods

### CAF01

The CAF01 adjuvant was manufactured as previously described by the thin-film hydration method (18). The two components of CAF01, composed of the quaternary ammonium compound dimethyldioctadecylammonium (DDA) and the glycolipid trehalose 6,6′-dibehenate (TDB), were synthetically manufactured by Avanti® Polar Lipids, AL, USA. In brief, weighed amounts of DDA and TDB (5:1) were dissolved in chloroform/methanol (9:1, v/v), and the organic solvent subsequently was removed by a rotary evaporator, thereby forming a thin lipid film at the bottom of the flask. The lipid film was rehydrated in Milli-Q water (Millipore, 18.2 MΩ cm at 25°C) with 10% trehalose (w/w) by heating for 20 min at 60°C with stirring. Freshly prepared CAF01 was freeze-dried in glass vials as previously described (22). The vials were frozen in dry ice, dissolved in acetone for 10 min, and placed on the plate of a freeze-dryer with a temperature of −40°C. At the end of the freeze-drying process, the glass vials containing CAF01 were stored at 4°C until use. The average zeta potential and size of CAF01 alone and complexed with RNA were measured at 25°C using a Nanosizer (Zetasizer Nano Series ZS, Malvern Instruments, UK) with disposable capillary cuvettes. The results are the average of triplicate independent measurements analyzed using Dispersion Technology Software (Malvern Instruments, UK).

### Generation of RNA Liposome Formulation

RNA was synthesized by *in vitro* transcription (IVT) using mMESSAGEmMACHINE™ SP6 Transcription Kit (Invitrogen), according to the manufacturer, from linearized DNA infectious clones. To evaluate RNA concentration from IVT, an RNA standard was made. IVT RNA for standard was purified using QIAamp® Viral RNA Mini Kit (QIAGEN) according to the manufacturer and concentration measured by NanoDrop™. Different concentrations of standard were run on a 1% agarose gel together with experimental IVT RNA. A visual approximation of the amount of RNA of interest was made and validated using ImageJ software. Purification of the alphavirus genome post IVT was found to reduce or ablate infectivity. For *in vivo* inoculations, 0.3 mg of CAF01 was added to virus production serum-free medium (VP-SFM) and vortexed at high speed for 5 s. Two micrograms of IVT RNA was added to the CAF01 VP-SFM solution to create a total volume of 20 μl for one dose. The formulation was mixed by vortexing at high speed for 5 s. The formulation was left at room temperature for 30 min with vortexing every 10 min prior to inoculation.

### Viruses and Cells

Vero cells were cultured in Opti-MEM (Gibco, Thermo Fisher Scientific, Australia), supplemented with 3% fetal calf serum (FCS). Virus and live-attenuated CHIKV-NoLS were propagated in Vero cells cultured in VP-SFM serum-free, ultra-low protein medium (Gibco, Thermo Fisher Scientific, Australia). RNA transfections carried out with Lipofectamine 2000 (Thermo Fisher Scientific, Australia) were performed according to the manufacturer's instructions. For CAF01 RNA transfections, 0.3 mg of CAF01 was added to 500 μl of VP-SFM (Gibco, Thermo Fisher Scientific, Australia) and vortexed at high speed for 5 s. One microgram of RNA was added to the CAF01 VP-SFM solution and mixed by vortexing at high speed for 5 s. The mixture was left at room temperature for 30 min with vortexing every 10 min. The reaction was added to confluent Vero cells that had been washed in phosphate-buffered saline (PBS). After 1 h, 500 μl of VP-SFM (Gibco, Thermo Fisher Scientific, Australia) was added to the reaction.

### Viral Titer Assay

Sera or media containing virus was added in triplicate to Vero cells. Virus was allowed to incubate for 1 h at 37°C in a 5% CO_2_ incubator before the virus was removed and the cells overlaid with Opti-MEM containing 3% FCS and 1% agarose (Thermo Fisher Scientific, Australia) and incubated for 48 h at 37°C in a 5% CO_2_ incubator. Cells were fixed in 1% formalin, and virus plaques were made visible by staining with 0.1% crystal violet. Results are expressed as plaque-forming units (pfu) per milliliter.

### Flow Cytometry

RNA transfected cells were gently resuspended and fixed in 4% paraformaldehyde. Cells were stained with LIVE/DEAD (Thermo Fisher Scientific, Australia) and capsid protein-specific antibody (12) with Alexa Fluor® 488 anti-mouse secondary antibody (Invitrogen, Thermo Fisher Scientific, Australia). Infectivity was measured as the number and percentage of 488-positive live cells using a BD LSR II Fortessa Cell Analyser and quantified with FlowJo software (v10.6; Treestar, Inc.).

Mice were sacrificed at day 6 post challenge and perfused intracardially with PBS, and feet collected. Bone marrow cells were flushed out using PBS, and feet were digested with collagenase IV (2 mg/ml, Worthington Biochemical Corporation) and DNase I (5 μg/ml, Sigma Aldrich) in RPMI and 10% FCS (Sigma Aldrich) and then filtered through 70- and 30-μm nylon meshes to obtain single-cell suspensions. Cells were stained with fluorochrome-conjugated antibodies against CD3 (clone 17A2, BioLegend), CD4 (clone GK1.5, BioLegend), CD8 (clone 53-6.2, BD Biosciences), CD45 (clone 30-F11, BD Biosciences), TCRβ (clone H57-597, eBiosciences), CD11b (clone M1/70, BD Biosciences), MHCII (M5/114, eBioscience), and LIVE/DEAD. Sphero™ Blank Calibration Particles (BD Biosciences) were used as counting beads. Cells were analyzed using flow cytometry on a BD LSR II Fortessa Cell Analyser. Flow cytometry data were analyzed using FlowJo software (v10.6; Treestar, Inc.).

### Mouse Experiments

C57BL/6 WT mice were obtained from the Animal Resources Center (Perth, Australia). AG129 mice were bred in-house. Twenty-eight-day-old male and female mice, in equal distribution, were inoculated with viable virus or vaccine particles (10^4^ pfu CHIKV-WT or CHIKV-NoLS), liposome-delivered RNA (2 μg of CHIKV-WT or CHIKV-NoLS RNA complexed with 0.3 mg of CAF01) or RNA alone (2 μg of CHIKV-WT or CHIKV-NoLS RNA) subcutaneously in a 20-μl volume in the ventral/lateral side of the right foot. The immunologically competent C57BL/6 mouse footpad model of acute CHIKV disease recapitulates the arthritis, tenosynovitis, and myositis associated with mononuclear cell infiltration seen in CHIKV-infected patients. Mock-inoculated mice received 20 μl of VP-SFM media alone or VP-SFM media containing 0.3 mg of CAF01. Mice were monitored for changes in weight and the development of disease signs every 24 h. CHIKV-induced footpad swelling was assessed by measuring the height and width of the perimetatarsal area of the hind foot, using Kincrome digital vernier calipers. Disease signs in inoculated AG129 mice were scored on a scale of 0–4 where 4 is immediate humane endpoint. Clinical scores were assessed as follows: 0 = no disease; 1 = hunched, ruffled fur; 2 = a score of 1 in addition to dehydration, lethargy, ocular exudates, and diarrhea; 3 = a score of 1 and/or 2 in addition to vocalization, modified gait, hind limb weakness, and being emaciated; 4 = neurological disease signs (tremors/seizures), splayed legs, circling, hyperexcitability, weight loss ≥15%, and dragging limbs. Mice were sacrificed by CO_2_ asphyxiation at endpoints. Mice were challenged with 10^4^ pfu CHIKV-WT subcutaneously in the ventral/lateral side of the right (ipsilateral) or left (contralateral) foot 30 days after immunization. All animal procedures and experiments were performed in accordance with the guidelines set out by the Griffith University Animal Ethics Committee.

### Plaque Reduction Neutralizing Titer Assay

Serum samples were heat inactivated for 30 min at 56°C. A twofold dilution series of serum was produced from an initial 1:10 dilution. CHIKV-WT of 30 pfu was added to diluted serum and incubated at 37 °C for 1 h. The virus–serum inoculum was added to Vero cells and incubated at 37 °C for 1 h. A 1% agarose overlay (diluted in 2% FCS DMEM with Pen/Strep) was added to cells. Cells were incubated at 37 °C for 48 h, and plaques stained with crystal violet.

### ELISA

ELISA microtiter plates (96-well MaxiSorp, Nunc) were coated with 50 μl of CHIKV, purified by ultracentrifugation. Purified virus stocks were diluted in carbonate buffer (Na_2_CO_3_, NaHCO_3_; pH 9.5) prior to coating at a concentration of 20,000 pfu/μl. Plates were incubated overnight at 4°C. The following day, the virus solution was removed, and 50 μl of blocking buffer [PBS, 0.05% Tween-20 (v/v),5% skim milk (w/v)] was added to each well and incubated for 2 h at 37°C. Mouse serum samples were inactivated for 30 min at 56°C before diluting 1:100 in wash buffer [PBS, 0.05% Tween-20 (v/v)]. A 1:100 dilution was found to be optimal after performing a twofold dilution series (1:50–1:400). Blocking buffer was removed, and 50 μl of diluted serum samples, together with hyperimmune positive and negative controls, was added to the wells. After 1 h of incubation at 37°C, plates were washed four times using wash buffer. Fifty microliters of secondary antibody, goat anti-mouse IgG horseradish peroxidase (HRP) (1:6,000) or goat anti-mouse IgM HRP (1: 800), was added to each well accordingly and incubated for 1 h at 37°C. Plates were washed five times with the wash buffer, and 50 μl of 3,3′,5,5′-tetramethylbenzidine (TMB) substrate was added per well. After incubation at room temperature in the dark for 15 min, 50 μl of 2 M H_2_SO_4_ was added to stop the reaction. Optical density (OD) values were obtained using a Bio-Rad plate reader at 450-nm wavelength.

### Statistical Analysis

Non-parametric Mann–Whitney *t*-test was used to analyze cellular infiltration by flow cytometry, virus titer, weight gain, and foot swelling. One-way ANOVA with Tukey posttests was used to analyze IgM and IgG titers. Neutralizing antibody titers were analyzed by Kruskal–Wallis test with Dunn's posttests. A *P* < 0.05 was considered to be significant.

## Results

### CAF01 Delivers Replication-Competent CHIKV RNA *in vitro*

**Figure 1:**
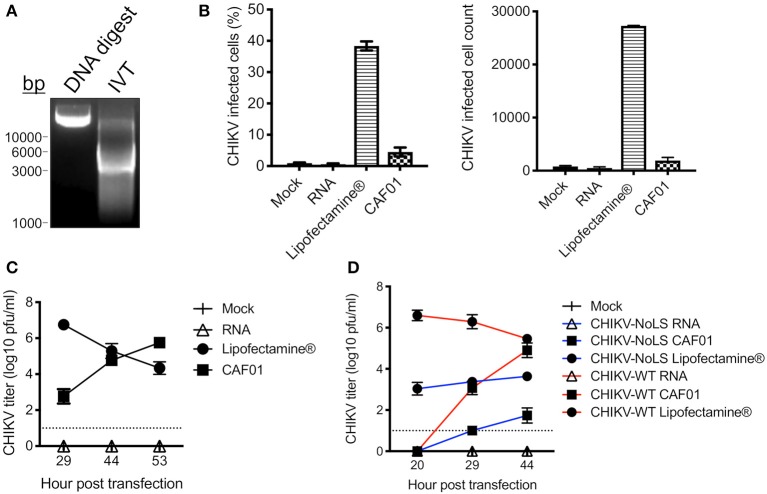
CAF01 RNA formulation and *in vitro* assays. **(A)** CHIKV-WT linearized DNA infectious clone (DNA digest) and *in vitro* transcribed CHIKV-WT RNA (IVT) were run on a 1% agarose gel and visualized using GelRed® under UV light. **(B)** CHIKV-WT RNA was transfected into Vero cells using either Lipofectamine® or CAF01. Cells were also mock transfected or transfected with RNA alone. At 12 h post transfection, infectivity (percentage and cell count) was measured by flow cytometry. **(C)** From 29 h post transfection, CHIKV-WT growth kinetics were analyzed by plaque assay. **(D)** CHIKV-WT and CHIKV-NoLS RNA was transfected into Vero cells using either Lipofectamine® or CAF01. Cells were also mock transfected or transfected with RNA alone. Viral growth kinetics were analyzed by plaque assay. Each symbol represents the mean ± standard error from three independent experiments.

To overcome potential production limits for our CHIKV vaccine candidate (CHIKV-NoLS), we developed a liposomal approach to deliver the replication-competent RNA genome of the live-attenuated vaccine. As proof of principle, CHIKV-WT RNA was *in vitro* transcribed from infectious cDNA clones (Figure 1A) and packaged with CAF01 liposomes. The surface charge and average size of CAF01 complexed with RNA were measured by dynamic light scattering. CAF01 in VP-SFM solution had a surface charge (zeta potential) of 23.4 mV. CAF01 with 2 μg RNA in VP-SFM had a zeta potential of −21.8 mV. The average size of CAF01–RNA complexes was 616.9 ± 67.45 nm, with a polydispersity index of 0.62 ± 13.35.

CHIKV-WT CAF01 formulations and CHIKV-WT RNA, delivered alone or with Lipofectamine®, were incubated with Vero cells, and at 12 h post transfection, infectivity was measured by flow cytometry. Approximately 5% of CHIKV-WT CAF01-transfected cells were positive for CHIKV-WT infection (Figure 1B). Cells transfected with RNA alone showed no CHIKV infectivity and no signs of cytopathic effect (tested up to 53 h post transfection). Results demonstrate that CAF01 is able to deliver replication-competent CHIKV RNA *in vitro*.

Cell transfected with CHIKV-WT CAF01 began to show signs of cytopathic effect at 29 h post transfection (data not shown). To examine the productive replication of CHIKV following CHIKV-WT CAF01 transfection, growth kinetics were analyzed by plaque assay. CHIKV-WT CAF01 transfected cells produced live infectious CHIKV with increasing titer to 53 h post transfection (Figure 1C). With a higher infectivity following transfection, cells transfected with Lipofectamine®-delivered CHIKV-WT RNA showed signs of cytopathic effect and produced high CHIKV titers earlier than cells transfected with CHIKV-WT CAF01. Cells transfected with CHIKV-WT RNA alone produced no live virus.

To determine the capability of CAF01 liposome to deliver replication-competent RNA capable of producing live-attenuated CHIKV-NoLS vaccine *in vitro*, growth kinetics were examined in Vero cells following transfection. Like CHIKV-WT CAF01-transfected cells, CHIKV-NoLS CAF01 transfection produced live CHIKV-NoLS at 29 h post transfection with increasing titer thereafter (Figure 1D).

### CAF01 Delivery of CHIKV-NoLS RNA in AG129 Mice Elicits *de novo* Viral Particle Production *in vivo*

**Figure 2:**
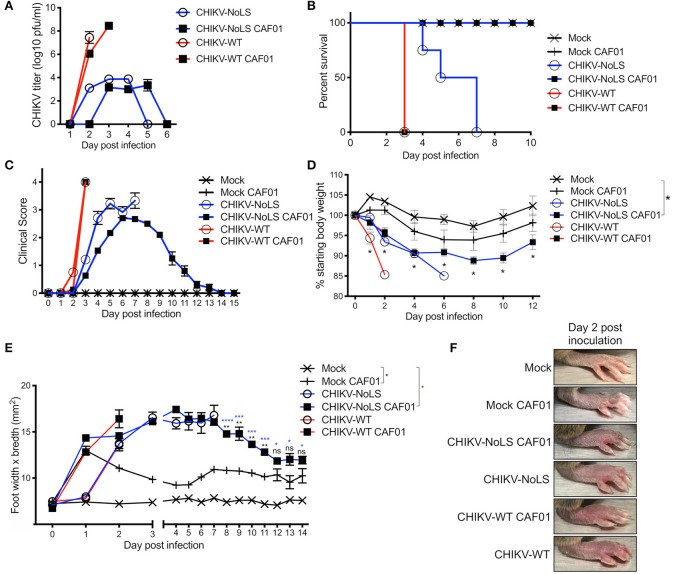
Inoculation with CHIKV-NoLS CAF01 elicits *de novo* viral particle production and disease in AG129 mice. AG129 mice were inoculated with viable virus/vaccine particles (10^4^ pfu CHIKV-WT or CHIKV-NoLS) or CAF01 delivered RNA (2 μg CHIKV-WT or CHIKV-NoLS RNA complexed with 0.3 mg CAF01) in the ventral/lateral side of the right foot. Mock inoculated mice received 20 μl VPSFM media or VPSFM media containing 0.3 mg CAF01. **(A)** Serum was collected on days 1–6 post inoculation and virus titrated by plaque assay. Survival curves were constructed **(B)** as mice were monitored for the development of disease signs **(C)** and changes in weight **(D)** every 24 h. Disease signs in inoculated AG129 mice were scored on a scale of 0–4 where 4 is humane endpoint. **(E,F)** CHIKV-induced footpad swelling was assessed by measuring the height and width of the perimetatarsal area of the foot, using Kincrome digital vernier calipers. Each symbol represents the mean ± standard error from five to six mice. Statistical analysis was performed by non-parametric Mann–Whitney *t*-test between the groups indicated on the figure legend by the bar and *color. ns, not significant,*P < 0.05, **P < 0.005, ***P < 0.001, and ****P < 0.0001.

Having demonstrated the RNA delivery capacity of CAF01 *in vitro*, we sought to determine the potential for CAF01 to deliver replication-competent RNA *in vivo*. To investigate *de novo* live-attenuated vaccine production *in vivo*, we first assessed CAF01 delivery of CHIKV-NoLS RNA in AG129 immunodeficient mice lacking type I and II interferon receptors. AG129 mice were inoculated with viable virus/vaccine particles (10^4^ pfu CHIKV-WT or CHIKV-NoLS) or CAF01-delivered RNA (2 μg CHIKV-WT or CHIKV-NoLS RNA complexed with 0.3 mg CAF01) in the ventral/lateral side of the foot. CHIKV-WT CAF01- and CHIKV-NoLS CAF01-inoculated mice develop detectable viremia (Figure 2A). Similar to CHIKV-WT infected mice, mice inoculated with CHIKV-WT CAF01 developed high-titer viremia by day 2 post inoculation. CHIKV-NoLS CAF01-inoculated mice developed a transient viremia with a similar profile to CHIKV-NoLS-inoculated mice, albeit with a day delay (Figure 2A). Results suggest CAF01 is able to deliver replication-competent CHIKV RNA *in vivo* to produce viable infectious CHIKV particles.

### AG129 Mice Inoculated With CHIKV-NoLS CAF01 Present With Disease Signs but Recover From Infection

As a lethal model of CHIKV infection, the attenuation of CHIKV-NoLS CAF01 was tested in AG129 mice. AG129 mice provide an ideal model to determine the virulence and infectivity of *de novo* synthesized virus resulting from CAF01 RNA delivery *in vivo*. AG129 mice were inoculated with viable virus particles (10^4^ pfu CHIKV-WT or CHIKV-NoLS) or CAF01-delivered RNA (2 μg of CHIKV-WT or CHIKV-NoLS RNA complexed with 0.3 mg of CAF01) subcutaneously in the ventral/lateral side of the foot. Mock-infected mice were inoculated with VP-SFM (vaccine production media) alone or VP-SFM containing 0.3 mg CAF01. Mice were monitored daily for clinical signs of disease (scored 0–4, where 4 is a humane endpoint), weight loss, and footpad swelling. CHIKV-WT and CHIKV-WT CAF01-infected mice reached endpoint by day 3 post infection (Figure 2B), suggesting that the CHIKV-WT virus produced *in vivo* by CHIKV-WT CAF01 inoculation is as virulent as CHIKV-WT delivered as virus particles at 10^4^ pfu. CHIKV-NoLS-infected mice reached endpoint between days 4 and 7 post infection (Figure 2B). Results further demonstrate the high degree of CHIKV-NoLS attenuation. CHIKV-NoLS CAF01-inoculated mice presented with disease signs (Figure 2C), including ruffled fur, significantly reduced weight gain compared to mock-inoculated mice (Figure 2D), and increased lethargy, but recovered from the inoculation. Unlike CHIKV-WT, CHIKV-NoLS, and CHIKV-WT CAF01-infected mice, mice inoculated with CHIKV-NoLS CAF01 presented with no neurological signs of disease to prompt endpoint.

CHIKV-WT, CHIKV-NoLS, CHIKV-WT CAF01, and CHIKV-NoLS CAF01-inoculated mice rapidly developed intense footpad swelling by day 2 post inoculation (Figures 2E,F). In CHIKV-NoLS, CAF01-inoculated mice, footpad swelling began to recover by day 5 post inoculation and resolved close to mock-infected levels, beginning to plateau, at day 13 post inoculation (Figure 2E). A degree of footpad swelling was observed in mock-CAF01-inoculated mice in the days following inoculation. This swelling was, however, minimal compared to that observed in other groups with no significant difference compared to mock-inoculated mice from day 12 post inoculation (Figure 2E).

### CHIKV-NoLS CAF01-Inoculated AG129 Mice Survive CHIKV-WT Challenge and Are Protected From Development of CHIKV Disease

**Figure 3:**
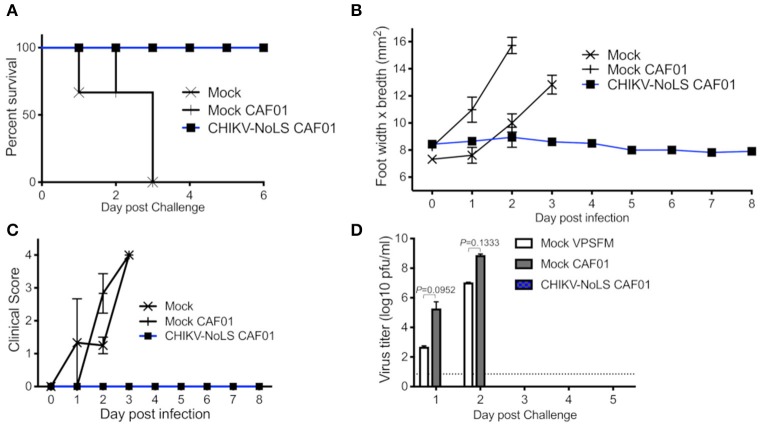
CHIKV-NoLS CAF01 inoculated AG129 mice survive CHIKV-WT challenge and are protected from development of disease. AG129 mice were inoculated with 2 μg CHIKV-NoLS RNA complexed with 0.3 mg CAF01 in the ventral/lateral side of the right foot. Mock inoculated mice received 20 μl VP-SFM media or VP-SFM media containing 0.3 mg CAF01. Mice were challenged with 10^4^ pfu CHIKV-WT subcutaneously in the ventral/lateral side of the right (ipsilateral) foot 30 days post inoculation. Survival curves were constructed **(A)** as mice were monitored for CHIKV-induced footpad swelling **(B)**, the development of disease signs **(C)** and changes in weight every 24 h. Disease signs in inoculated AG129 mice were scored on a scale of 0–4 where 4 is humane endpoint. **(D)** Serum was collected on days 1–5 post challenge and virus titrated by plaque assay. Each symbol represents the mean ± standard error from five to six mice. Statistical analysis was performed by non-parametric Mann–Whitney *t*-test.

As CHIKV-NoLS CAF01-inoculated AG129 mice survived, AG129 mice were used as a lethal challenge model to test the vaccine efficacy of CHIKV-NoLS CAF01. AG129 mice were inoculated with CHIKV-NoLS CAF01 or mock infected with VP-SFM alone or VP-SFM containing 0.3 mg of CAF01 subcutaneously in the ventral/lateral side of the foot. At 30 days post immunization, mice were challenged with 10^4^ pfu CHIKV-WT in the ventral/lateral side of the ipsilateral (immunized) foot and monitored daily for clinical signs of disease, weight loss, and footpad swelling. Mock-immunized mice reached endpoint between days 1 and 3 post challenge. Mice immunized with CHIKV-NoLS CAF01 survived the challenge (Figure 3A) with no signs of footpad swelling (Figure 3B) or clinical signs of disease (Figure 3C). Furthermore, CHIKV-NoLS CAF01-immunized mice developed no detectable viremia upon CHIKV-WT challenge (Figure 3D). These observations suggest immunization with CHIKV-NoLS CAF01 is capable of protecting AG129 mice from challenge for up to 30 days, indicating protective efficacy after a single dose.

### CHIKV-NoLS CAF01 Inoculation Protects Against CHIKV Disease in the Ipsilateral Foot of C57BL/6 Mice Challenged With CHIKV-WT

**Figure 4:**
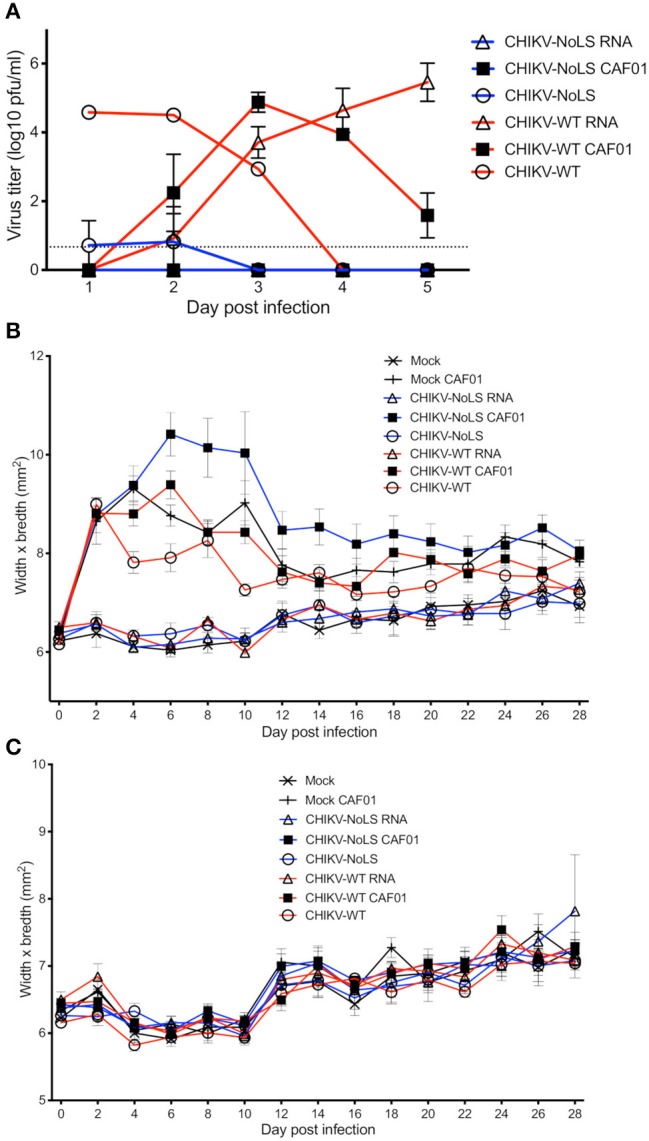
Viremia following CHIKV-NoLS CAF01 inoculation and CAF01 induced footpad swelling in C57BL/6 mice. C57BL/6 mice were inoculated with viable virus particles (10^4^ pfu CHIKV-WT or CHIKV-NoLS), CAF01 delivered RNA (2 μg CHIKV-WT or CHIKV-NoLS RNA complexed with 0.3 mg CAF01) or RNA alone (2 μg CHIKV-WT or CHIKV-NoLS RNA) subcutaneously in the ventral/lateral side of the right foot. Mock inoculated mice received 20 μl VP-SFM media alone or VP-SFM media containing 0.3 mg CAF01. **(A)** Serum was collected on days 1–5 post inoculation and virus titrated by plaque assay. Mice were monitored daily for signs of CHIKV-induced footpad swelling in the **(B)** ipsilateral (inoculated) and **(C)** contralateral (non-inoculated) foot. Each symbol represents the mean ± standard error from five to six mice.

To test CAF01 RNA delivery and the immunogenicity of CHIKV-NoLS CAF01 immunization in immunocompetent mice, C57BL/6 mice were inoculated with viable virus particles (10^4^ pfu CHIKV-WT or CHIKV-NoLS), CAF01-delivered RNA (2 μg of CHIKV-WT or CHIKV-NoLS RNA complexed with 0.3 mg of CAF01), or RNA alone (2 μg of CHIKV-WT or CHIKV-NoLS RNA) subcutaneously in the ventral/lateral side of the foot. Mock-infected mice were inoculated with VP-SFM (vaccine production media) alone or VP-SFM containing 0.3 mg of CAF01. Mice were monitored daily for signs of CHIKV-induced footpad swelling in the ipsilateral (immunized) and contralateral (non-immunized) foot. CHIKV-WT and CHIKV-NoLS developed a detectable viremia as seen previously (12). The viremia in CHIKV-WT CAF01 and CHIKV-WT RNA-inoculated mice developed more gradually and peaked at later times post inoculation compared to those infected with CHIKV-WT but produced similarly high titers (Figure 4A). Viremia in CHIKV-WT CAF01-inoculated mice peaked at day 3 post infection whereas viremia in CHIKV-WT RNA-inoculated mice continued to rise to day 5. The higher earlier titers in CHIKV-WT CAF01-inoculated mice may be responsible for the reduced viremia at day 5 compared to CHIKV-WT RNA. No viremia was detected in CHIKV-NoLS CAF01 or CHIKV-NoLS RNA-inoculated mice. Results demonstrate that inoculation of C57BL/6 mice with replication-competent CHIKV-WT RNA, in the presence or absence of CAF01, leads to *de novo* synthesis of virus particles *in vivo* and development of viremia.

Two peaks of footpad swelling were observed in CHIKV-WT-infected mice, at approximately days 2 and 8 post infection, as reported previously (Figure 4B) (12). As expected, CHIKV-NoLS-inoculated mice developed no footpad swelling (12, 13). Mock-infected mice and CHIKV-WT RNA- and CHIKV-NoLS RNA-inoculated mice also developed no footpad swelling. All mice inoculated with CAF01, including mock CAF01, developed a high degree of footpad swelling between days 2 and 12 post inoculation (Figure 4B). After this time, swelling began to resolve close to mock-infected levels, with near-complete resolution of swelling by day 28. No footpad swelling was observed in the contralateral foot of any group (Figure 4C).

**Figure 5:**
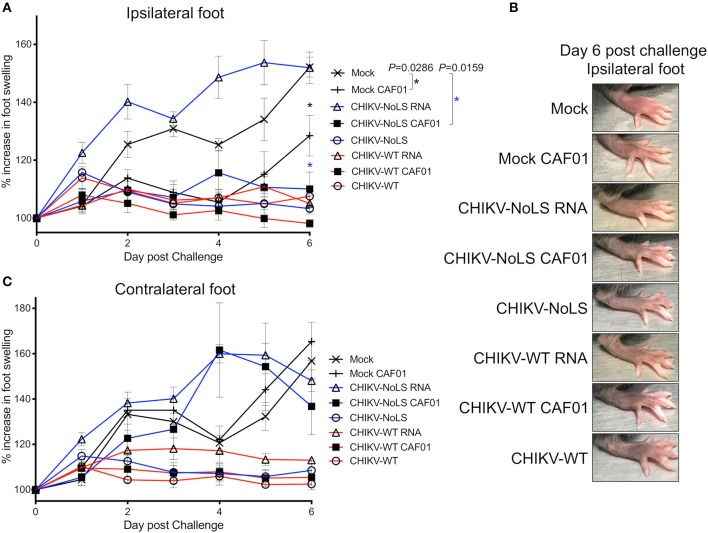
CHIKV-NoLS CAF01 inoculation protects against CHIKV disease in the ipsilateral foot of C57BL/6 mice upon CHIKV-WT challenge. C57BL/6 mice were inoculated with viable virus particles (10^4^ pfu CHIKV-WT or CHIKV-NoLS), CAF01 delivered RNA (2 μg CHIKV-WT or CHIKV-NoLS RNA complexed with 0.3 mg CAF01) or RNA alone (2 μg CHIKV-WT or CHIKV-NoLS RNA) subcutaneously in the ventral/lateral side of the right foot. Mock inoculated mice received 20 μl VP-SFM media or VP-SFM media containing 0.3 mg CAF01. At 30 days post inoculation, mice were challenged with 10^4^ pfu CHIKV-WT in the ventral/lateral side of the **(A,B)** ipsilateral or **(C)** contralateral foot and monitored daily for footpad swelling. Each symbol represents the mean ± standard error from five to six mice. Statistical analysis was performed by non-parametric Mann–Whitney *t*-test between the groups indicated on the figure legend by the bar and *color, and *P*-value.

At 30 days post inoculation, mice were challenged with 10^4^ pfu CHIKV-WT in the ventral/lateral side of the ipsilateral or contralateral foot and monitored daily for footpad swelling. Mice immunized with CHIKV-NoLS showed no signs of footpad swelling in either the contralateral or ipsilateral leg upon challenge with CHIKV-WT (Figures 5A–C). Results suggest that CHIKV-NoLS immunization provides systemic protection from CHIKV challenge after one dose. All mice inoculated with either CHIKV-WT, CHIKV-WT CAF01, or CHIKV-WT RNA similarly developed no footpad swelling in the contralateral or ipsilateral leg when challenged with CHIKV-WT (Figures 5A–C). In the ipsilateral and contralateral feet of mock-immunized mice challenged with CHIKV-WT, footpad swelling peaked at day 6 post challenge (Figures 5A–C).

Interestingly, CHIKV-WT challenge in mice immunized with CHIKV-NoLS RNA resulted in an earlier onset of foot swelling in both the ipsilateral and contralateral feet (Figures 5A,C). Early onset of disease swelling was also observed in the challenged contralateral foot of CHIKV-NoLS CAF01-immunized mice, peaking at days 4 and 5 post challenge (Figure 5C). However, the ipsilateral foot of CHIKV-NoLS CAF01-immunized mice developed no swelling when challenged with CHIKV-WT (Figure 5A). Swelling was also reduced at day 6 post challenge in the ipsilateral foot of mock-CAF01-immunized mice challenged with CHIKV-WT compared to mock-immunized mice, although to a lesser degree than CHIKV-NoLS CAF01-immunized mice. A discernible increase in swelling was observed at day 6 post challenge in the ipsilateral foot of mock CAF01-immunized mice compared to CHIKV-NoLS CAF01-immunized mice. Results suggest CHIKV-NoLS CAF01 immunization provides local but not systemic protection from CHIKV challenge.

### Cellular Infiltration in Challenged C57BL/6 Mice

**Figure 6:**
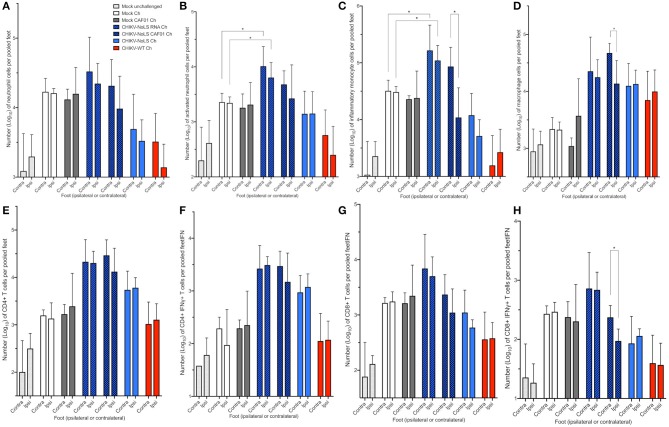
Cellular infiltration in challenged C57BL/6 mice. C57BL/6 mice were inoculated with viable virus particles (10^4^ pfu CHIKV-WT or CHIKV-NoLS), CAF01 delivered RNA (2 μg CHIKV-NoLS RNA complexed with 0.3 mg CAF01), or RNA alone (2 μg CHIKV-NoLS RNA) subcutaneously in the ventral/lateral side of the right foot. Mock inoculated mice received 20 μl VP-SFM media alone or VP-SFM media containing 0.3 mg CAF01. At 30 days post inoculation, all groups except Mock unchallenged mice were challenged (Ch) with 10^4^ pfu CHIKV-WT in the ventral/lateral side of the ipsilateral or contralateral foot and monitored daily for footpad swelling. Feet at 6 days post challenge were processed for flow cytometry analysis. The number of neutrophils **(A)**, activated neutrophils **(B)**, inflammatory monocytes **(C)**, macrophages **(D)**, CD4^+^ **(E)**, CD4^+^IFNγ^+^ **(F)**, CD8^+^ **(G)**, and CD8^+^IFNγ^+^ T cells **(H)** was examined. Each symbol represents the mean ± standard error from five to six mice. Statistical analysis was performed by non-parametric Mann–Whitney *t*-test. *P < 0.05.

To further understand the immune mechanisms responsible for early-onset disease and the local protective effect of CHIKV-NoLS CAF01 inoculation, flow cytometry was used to determine immune cell infiltration in the feet. To determine whether CHIKV-NoLS CAF01 provides local or systemic protection after CHIKV-WT challenge, mice were inoculated in the ipsilateral foot with CHIKV-NoLS CAF01 and subsequently challenged 30 days later with CHIKV-WT in both the ipsilateral and contralateral feet. The composition of immune cell infiltrates was assessed at day 6 post challenge in the ipsilateral and contralateral feet. The number of CD11b^+^ Ly6G^+^ neutrophils (Figure 6A), Ly6G^+^SSC^hi^ activated neutrophils (Figure 6B), CD11b^+^ Ly6C^hi^ inflammatory monocytes (Figure 6C), CD11b^+^ Ly6C^lo^ macrophages (Figure 6D), and CD4^+^ (Figure 6E), CD4^+^IFNγ^+^ (Figure 6F), CD8^+^ (Figure 6G), and CD8^+^IFNγ^+^ T cells (Figure 6H) was examined. CHIKV-NoLS RNA inoculated mice, which displayed early onset of severe swelling when challenged with CHIKV-WT, showed a marked increase in all cell types in both the ipsilateral and contralateral feet compared to mock-infected and challenged controls. The number of activated neutrophils and inflammatory monocytes was significantly higher in both ipsilateral and contralateral feet of CHIKV-NoLS RNA-inoculated mice compared to mock-infected controls following CHIKV challenge (Figures 6B,C). For both activated neutrophils and inflammatory monocytes, the number of cells in unchallenged controls was low and similar to cell numbers in the CHIKV-WT-inoculated group, which was protected from development of swelling upon CHIKV challenge. This suggests that activated neutrophils and inflammatory monocytes are involved in local CHIKV-induced inflammation upon challenge. That activated neutrophil and inflammatory monocyte cell numbers were high in both mock-challenged groups compared to unchallenged controls also emphasizes their involvement in CHIKV disease. Together, these data suggest that cellular infiltrates, particularly activated neutrophils and inflammatory monocytes, are involved in the early onset of severe CHIKV disease in CHIKV-NoLS RNA-challenged mice.

Significant differences in the cell number of inflammatory monocytes, macrophages, and CD8^+^IFNγ^+^ T cells were also observed between the feet of CHIKV-NoLS CAF01-inoculated mice following the challenge (Figures 6C,D,H). The contralateral foot had higher numbers of cells than the ipsilateral foot, suggesting that inflammatory monocytes, macrophages, and CD8^+^IFNγ^+^ T cells play a role in the rapid disease onset observed in the contralateral foot of CHIKV-NoLS CAF01-inoculated mice challenged with CHIKV.

### Antibody Response in CHIKV-NoLS CAF01-Inoculated C57BL/6 Mice

**Figure 7:**
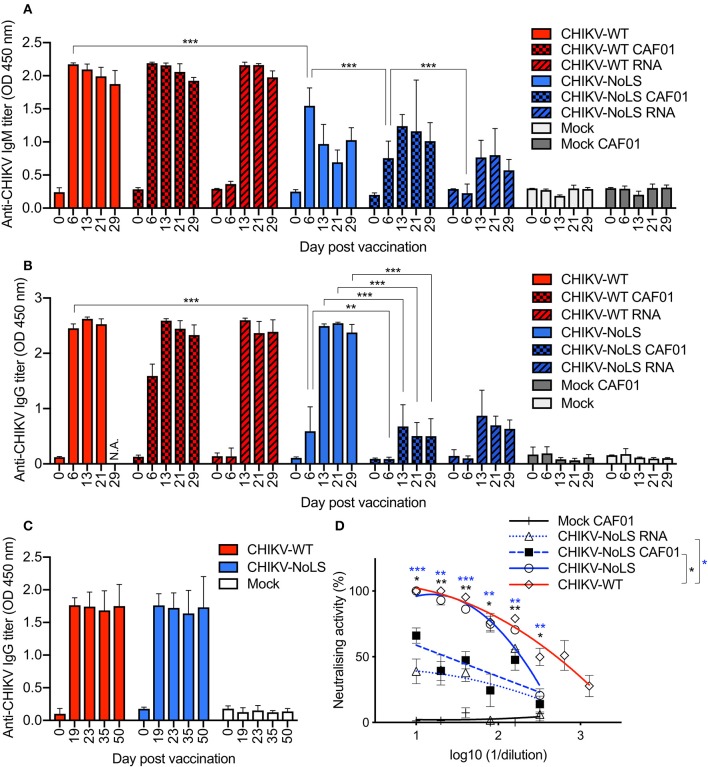
Antibody response in CHIKV-NoLS CAF01 inoculated C57BL/6 mice. C57BL/6 mice were inoculated with viable virus particles (10^4^ pfu CHIKV-WT or CHIKV-NoLS), CAF01 delivered RNA (2 μg CHIKV-WT or CHIKV-NoLS RNA complexed with 0.3 mg CAF01), or RNA alone (2 μg CHIKV-WT or CHIKV-NoLS RNA) subcutaneously in the ventral/lateral side of the right foot. Mock inoculated mice received 20 μl VP-SFM media or VP-SFM media containing 0.3 mg CAF01. Serum was harvested at various times post inoculation and titrated for IgM **(A)** and IgG **(B,C)** by ELISA. **(D)** Neutralizing antibody titers at day 30 post inoculation were examined. Each symbol represents the mean ± standard error from 5 to 6 mice. Statistical significance of IgM and IgG titers was analyzed by one-way ANOVA with Tukey posttests and neutralizing antibody titers by Kruskal–Wallis test with Dunn's posttests (groups indicated on the figure legend by the bar and *color). *P < 0.05, **P < 0.005, and ***P < 0.001. N.A., data not available.

CHIKV vaccine studies in mice and non-human primates indicate that neutralizing antibodies are the primary mediator of protection (23–25). We examined the humoral immunogenicity of CHIKV-NoLS CAF01 in mice receiving a single vaccination, as described above, using virion-based ELISA and plaque reduction neutralization assay. All mice inoculated with virus particles, CAF01-delivered RNA, or RNA alone seroconverted after one dose (Figures 7A,B). A significant difference between the IgM levels stimulated by CHIKV-NoLS and CHIKV-NoLS CAF01 was observed at day 6 post inoculation, with higher levels in CHIKV-NoLS-inoculated mice suggesting a more rapid IgM response (Figure 7A). IgM levels peaked at day 6 in CHIKV-NoLS-inoculated mice, whereas in CHIKV-NoLS CAF01-inoculated mice, IgM levels peaked at day 13 post inoculation. IgM was first detected in CHIKV-NoLS RNA-inoculated mice at day 13 post inoculation, suggesting a delayed IgM response in these mice. CAF01 stimulates a more rapid IgM response than delivery of RNA alone, with almost no IgM detected at day 6 post inoculation in both CHIKV-WT RNA and CHIKV-NoLS RNA compared to higher levels in CHIKV-WT CAF01 and CHIKV-NoLS CAF01 (Figure 7A). IgG titers in CHIKV-NoLS-inoculated mice were detected by day 6 post inoculation (Figure 7B). CHIKV-NoLS-inoculated mice maintained a high level of IgG, similar to that observed in CHIKV-WT-infected mice, at days 13, 21, and 29 post inoculation (Figure 7B). CHIKV-NoLS CAF01-inoculated mice developed only low levels of IgG, similar to CHIKV-NoLS RNA-inoculated mice (Figure 7B).

As a single inoculation of CHIKV-NoLS produced levels of IgG similar to those seen in CHIKV-WT-infected mice, the IgG response at later times post CHIKV-NoLS inoculation was examined in separate experiments. No difference in the IgG levels between CHIKV-WT- and CHIKV-NoLS-inoculated mice was observed up to day 50 post inoculation, demonstrating that the strong IgG response to one dose of CHIKV-NoLS is maintained at later times post inoculation (Figure 7C).

Inoculation of mice with CHIKV-NoLS vaccine formulations induced antibodies that were able to neutralize CHIKV-WT. Plaque reduction neutralizing antibody titer (PRNT) at day 30 post inoculation was expressed as the reciprocal of the serum dilution yielding a >80% (PRNT_80_) or >50% (PRNT_50_) reduction in the number of plaques. As a positive control, CHIKV-WT-infected mice produced PRNT_50_ of 507 and a PRNT_80_ of 94 (Figure 7D). No significant difference in the amount of neutralizing antibodies produced by CHIKV-NoLS or CHIKV-WT inoculation was observed. However, CHIKV-NoLS CAF01 and CHIKV-NoLS RNA inoculation produced significantly lower amounts of neutralizing antibody compared to CHIKV-WT inoculation. CHIKV-NoLS inoculation produced a PRNT_50_ of 193 and a PRNT_80_ of 71 (Figure 7D). CHIKV-NoLS CAF01 inoculation produced a PRNT_50_ of 24 (Figure 7D). CHIKV-NoLS RNA inoculation did not produce any PRNT_50_ antibodies.

## Discussion

The use of this novel liposome delivery system has a number of potential benefits over a traditional live-attenuated vaccine: (1) CHIKV-NoLS is currently passaged in Vero cells, an acceptable vaccine substrate, using VP-SFM, a serum-free, ultra-low-protein medium, and administered *in vivo* as a live-attenuated virus particle. Despite previous studies demonstrating attenuated replication is maintained after serial passage, extended *in vitro* passage of an RNA virus such as CHIKV-NoLS, with no proofreading machinery, could lead to unwanted mutations in the virus genome and the potential to revert to a WT phenotype (13). Bypassing *in vitro* propagation of CHIKV-NoLS by using liposomes to deliver CHIKV-NoLS RNA directly *in vivo*, initiating CHIKV-NoLS replication and particle production, would reduce the risk of accumulating deleterious mutations in CHIKV-NoLS and so reduce the risk of reversion to a WT phenotype. (2) As propagation of CHIKV-NoLS is limited by its attenuated replication, liposomal delivery of CHIKV-NoLS RNA would remove this inefficient production step and eliminate tissue culture-related by-product contamination. (3) CHIKV-NoLS particles are currently stored at −80°C to remain viable. Liposome/CHIKV-NoLS RNA formulations can be stored as a lyophilized powder at 2–8°C and reconstituted prior to immunization. Being able to store a vaccine this way is hugely cost-effective and would give target populations greater access to this vaccine. (4) Using adjuvanted liposomes, such as CAF01, which induces antigen-presenting cells, to deliver CHIKV-NoLS RNA will further promote a broad and long-lived immune response. Delivery of CHIKV-NoLS RNA using CAF01 has the potential to reduce the overall infectious vaccine dose while maintaining or improving the protective immune response to CHIKV-NoLS.

RNA has emerged as an effective platform to safely and cheaply deliver vaccines using delivery vehicles such as liposomes (14). Where DNA-based vaccine approaches risk chromosomal integration and potential mutagenesis, RNA vaccines overcome this threat (26). A number of promising RNA-based therapeutic and vaccine strategies have been recently developed to combat arboviruses using lipid delivery techniques (14, 27). To our knowledge, this is the first study to describe the use of CAF01 as an RNA delivery vehicle. Furthermore, our approach of delivering the replication-competent RNA genome of a live-attenuated vaccine candidate using CAF01 is highly novel. CAF01 has been shown to be a safe and tolerable approach to vaccine delivery in clinical trials (21). CAF01 successfully delivered replication-competent RNA *in vitro*, albeit at reduced efficiency compared to transfection reagent Lipofectamine®, which is optimized for *in vitro* use and thus not an ideal benchmark for clinical application. Subsequent replication of infectious vaccine is able to overcome the initial low transfection efficiency of CAF01, to potentially increase antigenicity *in vivo*.

A degree of footpad swelling was observed in AG129 and C57BL/6 mice inoculated with CAF01 alone. It is likely that the glycolipid immunomodulator adjuvant of CAF01 stimulates a local pro-inflammatory response at the site of inoculation, giving rise to the observed swelling. Such pro-inflammatory reactions have been observed in other vaccination strategies upon the addition of an adjuvant (28). Furthermore, it is likely that the local inflammatory response caused by CAF01 inoculation is pronounced in these studies due to the route of inoculation in the footpad. A local inflammatory response may not be have been detected with an alternate route of inoculation, for example, intramuscular. Indeed, vaccination with CAF01 in clinical trials did not cause local or systemic adverse effects besides transient soreness at the injection site (21).

Interestingly, CHIKV-NoLS CAF01-inoculated C57BL/6 mice, although protected from CHIKV disease when challenged at the site of inoculation, did not develop systemic protection from CHIKV challenge. In fact, an early onset of severe disease was seen in these mice when challenged in the contralateral foot. A similar disease profile was observed in the contralateral and ipsilateral feet of CHIKV-NoLS RNA-inoculated mice. This enhancement of inflammation has been observed in previous CHIKV vaccination strategies and likened to a phenomenon known as antibody-dependent enhancement (ADE) (25, 29). ADE occurs when antibodies with sub-neutralizing capacity enhance infectivity and disease severity rather than provide protection. In CHIKV-infected mice, higher viral RNA loads and more severe joint inflammation have been observed in the presence of sub-neutralizing concentrations of CHIKV-specific antibodies, demonstrating the role of antibody-mediated enhancement during CHIKV infection and pathogenesis (30). With significantly low levels of IgG in CHIKV-NoLS CAF01- compared to CHIKV-NoLS-inoculated mice, as well as reduced neutralizing antibodies, it is possible that antibody-mediated enhancement is responsible for the early onset of severe swelling seen in CHIKV-NoLS CAF01 and CHIKV-NoLS RNA-challenged mice. The kinetics of infection upon challenge in the ankle tissue and serum require further investigation to fully demonstrate an ADE-like effect. However, these important observations reveal the need to assess vaccine candidates for the potential to cause disease enhancement, particularly vaccines that require booster regimes and where compliance in completing a vaccination booster schedule may be an issue. Vaccine strategies such as CHIKV-NoLS, and other live-attenuated vaccine candidates, that elicit a long-lasting IgG response after one dose have the potential to overcome these hurdles.

The protective effect of CHIKV-NoLS CAF01 inoculation in the ipsilateral foot upon CHIKV-WT challenge likely involves the local inflammatory response stimulated by the CAF01 glycolipid adjuvant during vaccination. Results also suggest that immune mechanisms, in addition to neutralizing antibodies, mediate local protection from CHIKV disease in mice. Further studies are ongoing to evaluate the systemic protective effect of CHIKV-NoLS CAF01 using prime boost strategies and more clinically relevant sites of inoculation, including intramuscular sites.

## Data Availability Statement

The raw data supporting the conclusions of this article will be made available by the authors, without undue reservation, to any qualified researcher.

## Ethics Statement

The animal study was reviewed and approved by The Griffith University Animal Ethics Committee.

## Author Contributions

MZ and AT: conceptualization and visualization. EA, MZ, and AT: methodology. EA, JF, AZ, MZ, and AT: validation. EA, KT, JF, HM, AZ, MZ, and AT: formal analysis. EA, JF, AZ, and AT: investigation. SM, AZ, MZ, and AT: resources. EA, AZ, MZ, and AT: writing—original draft preparation. EA, SM, AZ, MZ, and AT: writing—review and editing. SM, AZ, and AT: supervision. AT: project administration and funding acquisition. All authors contributed to manuscript revision and read and approved the submitted version.

### Conflict of Interest

The authors declare that the research was conducted in the absence of any commercial or financial relationships that could be construed as a potential conflict of interest.
